# Promising Thermoelectric Performance in Two-Dimensional Semiconducting Boron Monolayer

**DOI:** 10.3389/fchem.2021.739984

**Published:** 2021-09-22

**Authors:** Yonglan Hu, Ding Li, Rongkun Liu, Shichang Li, Chunbao Feng, Dengfeng Li, Guangqian Ding

**Affiliations:** School of Science, Chongqing University of Posts and Telecommunications, Chongqing, China

**Keywords:** boron monolayer, thermoelectric, first-principles, Boltzmann, phonon

## Abstract

A heavy element is a special character for high thermoelectric performance since it generally guarantees a low lattice thermal conductivity. Here, we unexpectedly found a promising thermoelectric performance in a two-dimensional semiconducting monolayer consisting of a light boron element. Using first-principles combined with the Boltzmann transport theory, we have shown that in contrast to graphene or black phosphorus, the boron monolayer has a low lattice thermal conductivity arising from its complex crystal of hexagonal vacancies. The conduction band with an intrinsic camelback shape leads to the high DOS and a high *n*-type Seebeck coefficient, while the highly degenerate valence band along with the small hole effective mass contributes to the high *p*-type power factor. As a result, we obtained the *p*-type thermoelectric figure of merit up to 0.96 at 300 K, indicating that the boron monolayer is a promising *p*-type thermoelectric material.

## Introduction

In the past decade, people devoted themselves to improve the thermoelectric efficiency by trying to individually control the thermoelectric coefficients through low-dimensional crystals such as single layers, nanowires, 2D heterostructures, and nanotubes. The thermoelectric performance of a material is usually characterized by the dimensionless figure of merit *ZT = S* ^*2*^ *σT/κ*, where *S* is the Seebeck coefficient, *σ* is the electrical conductivity, *κ* is the thermal conductivity including both electronic and lattice contributions, and *T* is the absolute temperature, respectively (Mohanraman et al., 2015; Hu et al., 2021). High ZT requires a superior electronic transport but a minimized phonon transport at the same time, the latter usually arises from heavy elements (Ding et al., 2016). For instance, the lattice thermal conductivity of traditional commercial bulk thermoelectric materials such as Bi_2_Te_3_ and PbTe are lower than 1 W/mK (Pei and Liu, 2012; Hellman and Broido, 2014). Although the thermoelectric coefficient in some cases can be individually controlled in a low-dimensional crystal, the high lattice thermal conductivity still prevents a striking improvement of *ZT* (Kumar and Schwingenschlögl, 2015).

Balandin et al. (2008) experimentally reported that the thermal conductivity of single-layer graphene is higher than 4000 W/mK at room temperature. For monolayer MoS_2_, it is about 100 W/mK at 300 K based on Yang’s report (Jin et al., 2015). Using a molecular dynamics simulation, Xu et al. (2015) obtained the lattice thermal conductivity of phosphorene along the zigzag direction that is higher than 150 W/mK at 300 K. Among these popular single-layer crystals, it was found that an extremely high thermal conductivity leads to poor *ZT*, which can be ascribed to the following two factors: 1) light elements with high vibration frequency and 2) large atomic weight difference forbids the anharmonic scattering. In this regard, we intended to think that is there possibility to achieve promising thermoelectric transport in other single-layer crystal consisting of light elements? In recent years, boron, one of the carbon’s nearest neighbors, demonstrated the polymorphism in two-dimensional crystals, which are called borophene. However, most of the boron monolayers were found to be metallic by experiment or theory. Interestingly, Hu *et al.* recently proposed a series of semiconducting boron monolayers formulated by the connected network of hexagonal vacancies (Xu et al., 2017). Such semiconducting phases of the boron monolayer are expected to achieve in experiments since the controlled synthesis of the boron monolayer is a mature technology (Kong et al., 2018; Kiraly et al., 2019).

The semiconducting $\beta_{1}^{s}$ boron monolayer has an indirect bandgap of 0.74 eV based on HSE06 functional (Xu et al., 2017), and the multi-valley character of both conduction and valence band near the Fermi level indicates the promising electronic transport performance. In addition, the complex crystal consisting of twelve boron atoms and hexagonal vacancies leads to large number of coupled phonon branches, which points to possible low lattice thermal conductivity in the crystal. To explore the potential of the semiconducting $\beta_{1}^{s}$ boron monolayer as a thermoelectric material, we studied its thermoelectric transport performance by first-principles combined with Boltzmann transport equations. We found that the lattice thermal conductivity is 20.2 W/mK at 300 K, and highly degenerate hole pockets with small effective mass lead to the high *p*-type power factor. Finally, the optimal *ZT* reaches 0.96 at 300 K for *p*-type doping, which is a recorded value among two-dimensional monolayers.

## Computational Details

The first-principles calculations were performed within the framework of density function theory (DFT) using projector-augmented wave (PAW) (Perdew et al., 1997) pseudopotentials and Perdew–Burke–Ernzerhof (PBE) (Kresse and Furthmüller, 1996) exchange correlation functionals as implemented in VASP (Tran and Blaha, 2009). To construct the single-layer crystal, a 15-Å-thick vacuum slab was added along the *z*-direction. The plane-wave cutoff energy was set to 400 eV and the Monkhorst–Pack ***k*** mesh was 15 × 15 × 1. Geometry optimization was converged until the force acting on the ions become smaller than 10^−3^ eV/Å. When we calculated the electronic structure, a modified Becke–Johnson (mBJ) (Tran and Blaha, 2009) functional was also considered to yield the accurate effective mass and bandgap.

The electronic transport properties were calculated using the Boltzmann transport equation (BTE) under a constant relaxation time approximation as implemented in BoltzTraP (Madsen and Singh, 2006). A rigid band approximation is used to treat doping, and the Fermi level shifts up for *n*-type doping while down for the *p*-type. However, within this approximation, the Seebeck coefficient can be calculated independent of carrier relaxation time *τ*, while the evaluation of electrical conductivity still requires the knowledge of *τ*. In this regard, we employed deformation potential theory based on effective mass approximation to calculate *τ* (Herring and Vogt, 1956). At last, we performed phonon BTE solution as implemented in the ShengBTE (Li et al., 2014) package to calculate lattice thermal conductivity. Second- and third-order interatomic force constants (IFCs) are quite necessary inputs for pBTE, which were obtained from DFT calculations using a converged 4 × 4 × 1 supercell. The phonon spectrum was obtained from the Phonopy code (Togo et al., 2008), and a converged cutoff distance of 0.4 nm for interactive distance was used in calculating anharmonic IFCs.

## Results and Discussion

**FIGURE 1:**
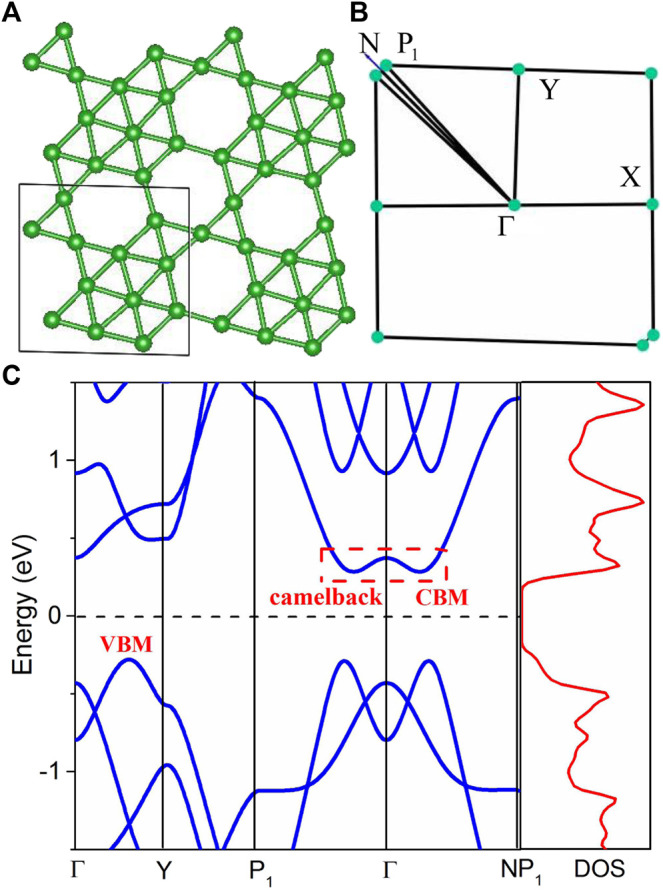
The atomic structure **(A)** and the Brillouin zone path **(B)** of the $\beta_{1}^{s}$ boron monolayer and **(C)** shows the calculated band structure and density of states.

Figure 1A shows the crystal structure of the $\beta_{1}^{s}$ semiconducting boron monolayer, which consists of a connected network of hexagonal vacancies that can be divided into triangle regions and heptagon regions, according to Hu *et al* (Xu et al., 2017). The space group is *Amm2* and the lattice parameter 6.12 Å after relaxation is consistent with Hu’s result (Xu et al., 2017). Figure 1B shows the Brillouin zone path, that is, Γ-Y-P_1_-Γ-N-P_1_. Based on Hu *et al* (Xu et al., 2017), the HSE06 band structure indicates that the $\beta_{1}^{s}$ boron monolayer is an indirect semiconductor with a bandgap of 0.74 eV, and also, the phonon spectrum and molecular dynamics simulation confirm the thermal stability of this boron monolayer. In contrast to monolayer TMDCs with a large bandgap, the moderate bandgap of the boron monolayer may possess better electronic transport performance.

As shown in Figure 1C, the calculated band structure of the $\beta_{1}^{s}$ boron monolayer displays an indirect bandgap of 0.68 eV based on mBJ modification, which is very close to the result of HSE06 (Xu et al., 2017). The mBJ functional has been shown to yield the accurate bandgap, effective mass, and frontier-band ordering. The conduction band minimum (CBM) is located in the interval between Γ and P_1_ points, while the valence band maximum (VBM) is located in the interval between Γ and Y points. In consistent with previous calculation, the VBM is dominated by the out-of-plane *p*_*z*_ orbitals while the CBM is attributed to the in-plane *s*+*p*_*x,y*_ orbitals (Xu et al., 2017), and this semiconducting $\beta_{1}^{s}$ boron monolayer was realized by modulating the in-plane *s*+*p*_*x,y*_ orbitals and *p*_*z*_-derived bands through the connected network of hexagonal vacancies, according to Hu *et al* (Xu et al., 2017).

Interestingly, the band structure of the $\beta_{1}^{s}$ boron monolayer possesses several advantages of electronic transport performance. First, the lowest conduction band, shown in Figure 1C, exhibits a camelback shape along the P_1_-Γ-N direction. The camelback shape is known in topological materials where the spin-orbital coupling is not large enough to cause inversion between the frontier bands (Eremeev et al., 2010). Here, this interesting band dispersion is obtained in the light $\beta_{1}^{s}$ boron monolayer. The importance of the camelback shape in electronic transport is that it can increase the number of degenerate carrier pockets, which thereby increases the density of states (DOS) effective mass (Ding et al., 2019a; Ding et al., 2019b). As one can see in the right panel of Figure 1C, the DOS at the CBM is markedly higher than that at VBM. As a result, a higher *n*-type Seebeck coefficient can be achieved in this boron monolayer. In addition to CBM, there are these band extremes of VBM along Γ-Y, P_1_-Γ, and Γ-N, respectively, which are highly degenerate in energy and indicate more carrier pockets joining in hole transport. The carrier effective mass near the Fermi level dominates the carrier mobility and relaxation time and plays an important role in thermoelectric transport (Peng et al., 2014). As one can see, the band near the VBM is more dispersive than that near the CBM, yielding a hole effective mass 0.57 *m*_0_ smaller than the 0.998 *m*_0_ of an electron.

**FIGURE 2:**
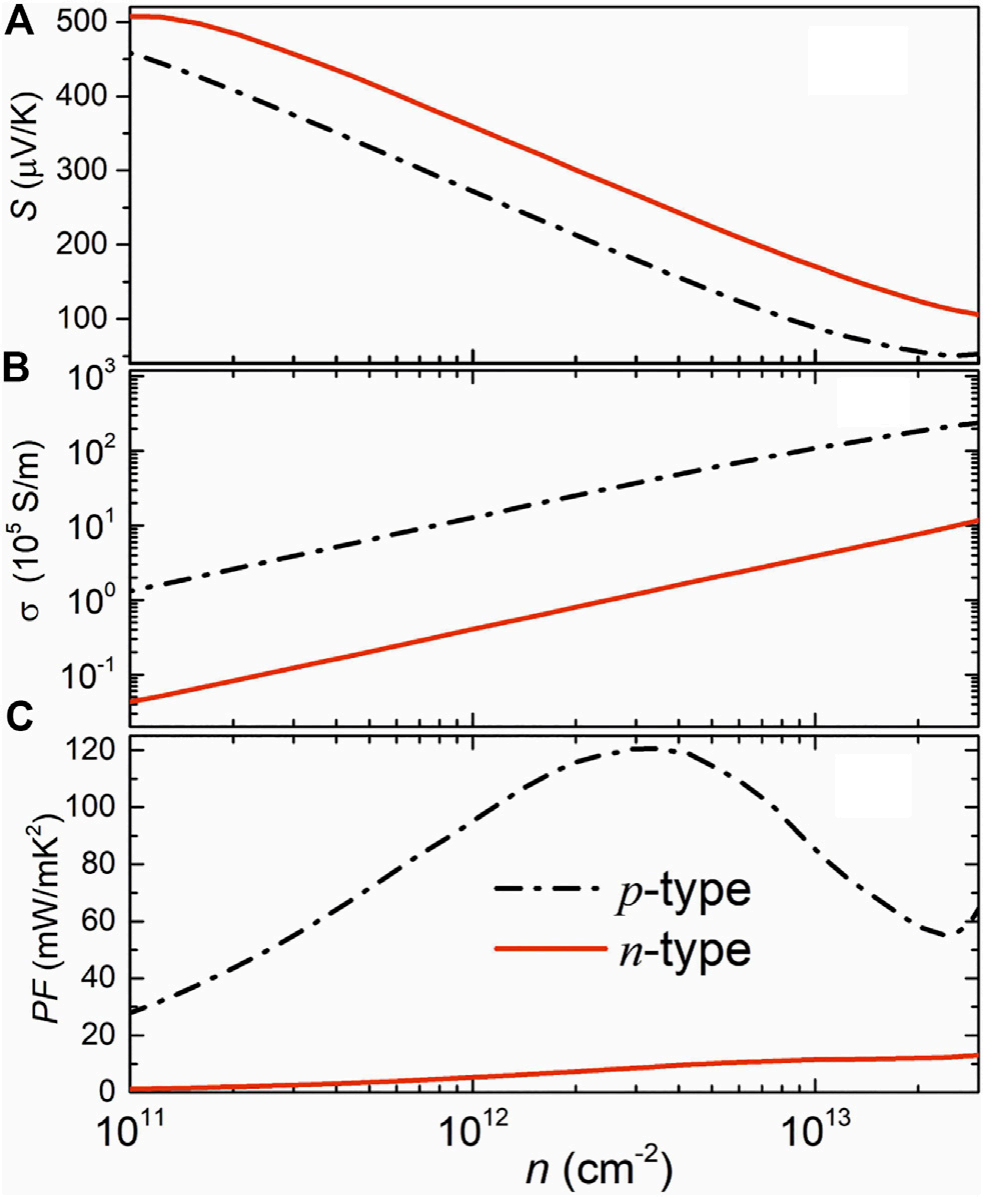
Calculated Seebeck coefficient **(A)**, electrical conductivity **(B)**, and the power factor **(C)** of the $\beta_{1}^{s}$ boron monolayer at 300 K.

Calculated electronic transport properties including the Seebeck coefficient, electrical conductivity, and the power factor at room temperature are shown in Figure 2. The Seebeck coefficient decreases while the electrical conductivity increases with the increase of carrier density since they are inversely related to carrier density. In this regard, the power factor cannot be improved infinitely but can be optimized by modulating carrier density. In Figure 2A, the higher *n*-type Seebeck coefficient can be attributed to the camelback-shaped band, as discussed above. To obtain the electrical conductivity as shown in Figure 2B, we employed deformation potential theory (Herring and Vogt, 1956) to calculate the carrier relaxation time. Calculated results are shown in Table 1. It is crucial to find that the lower deformation potential constant of holes reflects the minimal sensitivity of valence band maximum to deformation. Along with the smaller hole effective mass, a high hole mobility and long hole relaxation time were obtained, as compared to an electron. As a result, the *p*-type power factor is much higher than *n*-type, as shown in Figure 2C. The maximum power factor of the *p*-doped semiconducting boron monolayer reaches 121 mW/mK^2^ at an optimal carrier density of 3.14 × 10^12^ cm^−2^, while it is only about 17mW/mK^2^ in monolayer MoS_2_ (Jin et al., 2015). The Seebeck coefficient under this optimal carrier density for *n*- and *p*-type are 263 µV/K and 175 µV/K, respectively, which are the standard values of thermoelectric materials (Sun and Singh, 2016).

**TABLE 1:** Calculated DP constant, elastic modulus, carrier effective mass, carrier mobility, and carrier relaxation time at 300 K.

	*E*_*l*_	*C*_*2D*_	m*	μ	τ
	(eV)	(eVÅ⁻^2^)	(*m_e_*)	(cm^2^V1⁻^1^s⁻^1^)	(10⁻^14^s)
*n*-type	−6.93	29.2	0.998	208.6	11.797
*p*-type	−3.03	29.2	0.57	3,344.7	108.034

**FIGURE 3:**
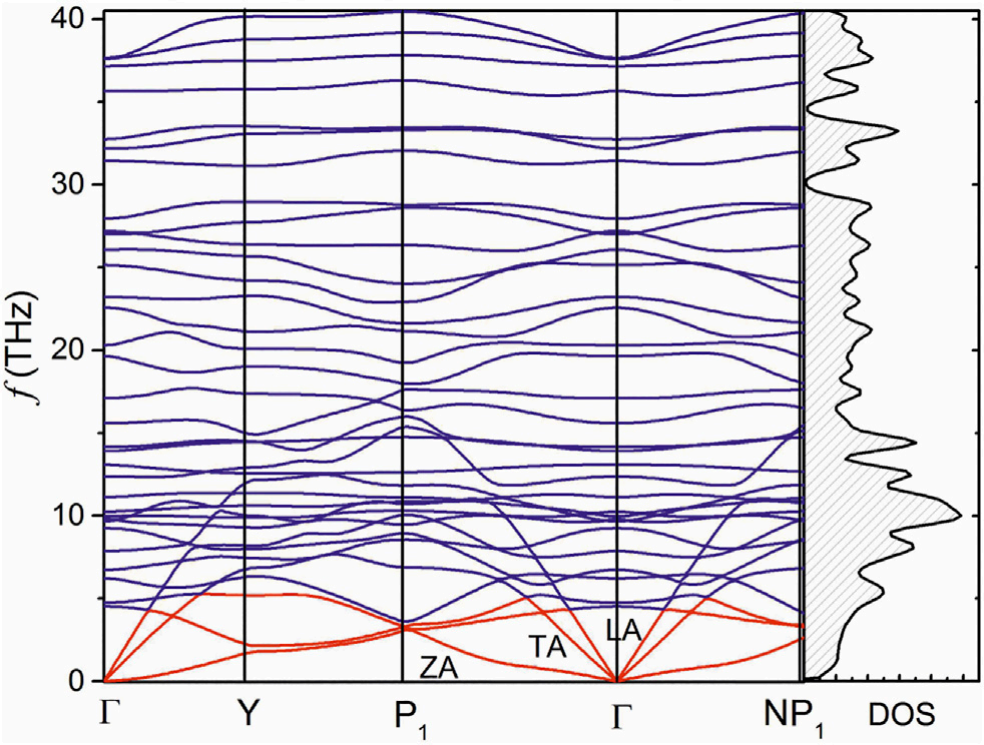
The phonon dispersion and density of states of the $\beta_{1}^{s}$ boron monolayer.

Calculated phonon dispersion of the $\beta_{1}^{s}$ boron monolayer is shown in Figure 3. First, in contrast to graphene, in phosphorene and monolayer MoS_2_, the twelve atoms in the unit cell leads to thirty-six phonon branches. It was found that a complex crystal with many optical modes is usually associated with low lattice thermal conductivity (Ding et al., 2018; Hu et al., 2020a; Hu et al., 2020b). A number of optical modes gather in frequency about 10 THz. High-frequency phonons with low velocity often do little contribution to lattice thermal conductivity. One can also see that the low-lying optical modes are coupled with acoustic modes, which is different from phosphorene or monolayer MoS_2_ where there is a wide frequency gap among optical branches or between acoustic and optical branches (Fei et al., 2014; Jin et al., 2015). A strong coupling of phonon modes will increase the anharmonic scattering processes and leads to the low lattice thermal conductivity. Although the allowed phonon frequency of about 40 THz is higher than that of phosphorene and monolayer MoS_2_ due to the light element, the allowed acoustic frequency of about 5 THz of phonon modes is quite lower than graphene, phosphorene, and monolayer MoS_2_ (Fei et al., 2014; Jin et al., 2015; Ge et al., 2016). These advantages of low lattice thermal conductivity in the $\beta_{1}^{s}$ boron monolayer are probably associated with its complex crystal of hexagonal vacancies.

**FIGURE 4:**
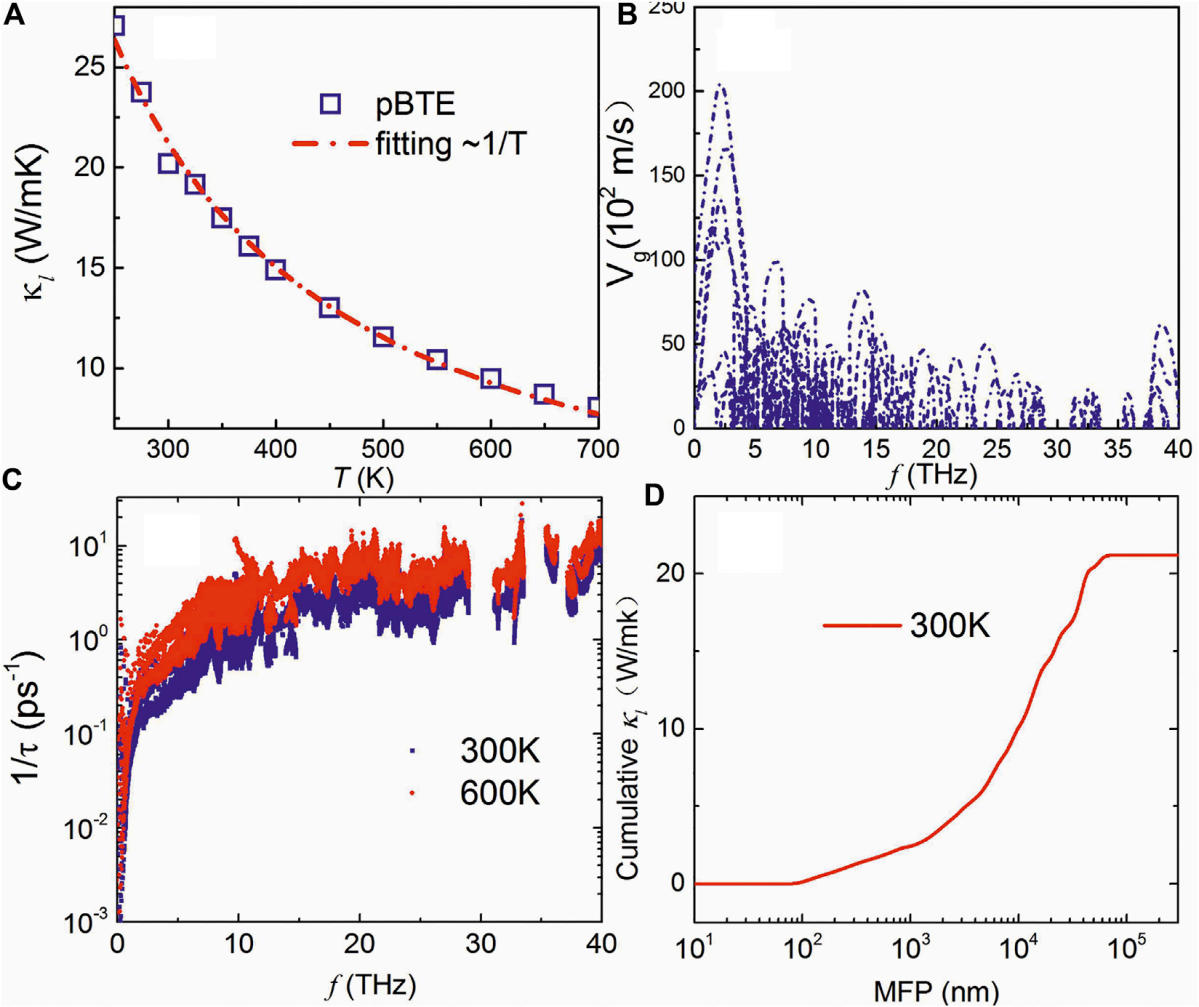
**(A)** Calculated lattice thermal conductivity with respect to temperature, phonon group velocity **(B)**, and anharmonic scattering **(C)** of the $\beta_{1}^{s}$ boron monolayer. **(D)** shows the cumulative lattice thermal conductivity with respect to the mean free path at 300 K.

Figure 4A shows the calculated lattice thermal conductivity of the boron monolayer with respect to temperature. It can be seen that the BTE results are well fitted with κ∝1/T. The lattice thermal conductivity at room temperature is about 20 W/mK, which is much lower than that of graphene (above 4000 W/mK) (Balandin et al., 2008), phosphorene (above 150 W/mK along zigzag) (Xu et al., 2015) consisting of light element, and also monolayer MoS_2_ (about 100 W/mK) (Jin et al., 2015). Thus, the $\beta_{1}^{s}$ boron monolayer with a light boron element in the crystal also exhibits low lattice thermal conductivity, as compared to previous light monolayers. Such a low lattice thermal conductivity can be ascribed to the large number of optical modes and the strongly coupled phonon modes as arising from the complex unit cell with a network of hexagonal vacancies, as discussed above. The low group velocity of optical modes, as shown in Figure 4B, indicates that the acoustic and low-lying optical modes do most of the contribution to phonon transport. One can see from Figure 4C that the anharmonic scattering rate increases with the increase in temperature, and such scattering rate is much higher than monolayer MoS_2_ in which the scattering rates of phonons almost lie below 1ps^−1^ (Ding et al., 2018). Figure 4D shows the cumulative lattice thermal conductivity as a function of mean free path at 300 K, which points to the well convergence of the lattice thermal conductivity.

**FIGURE 5:**
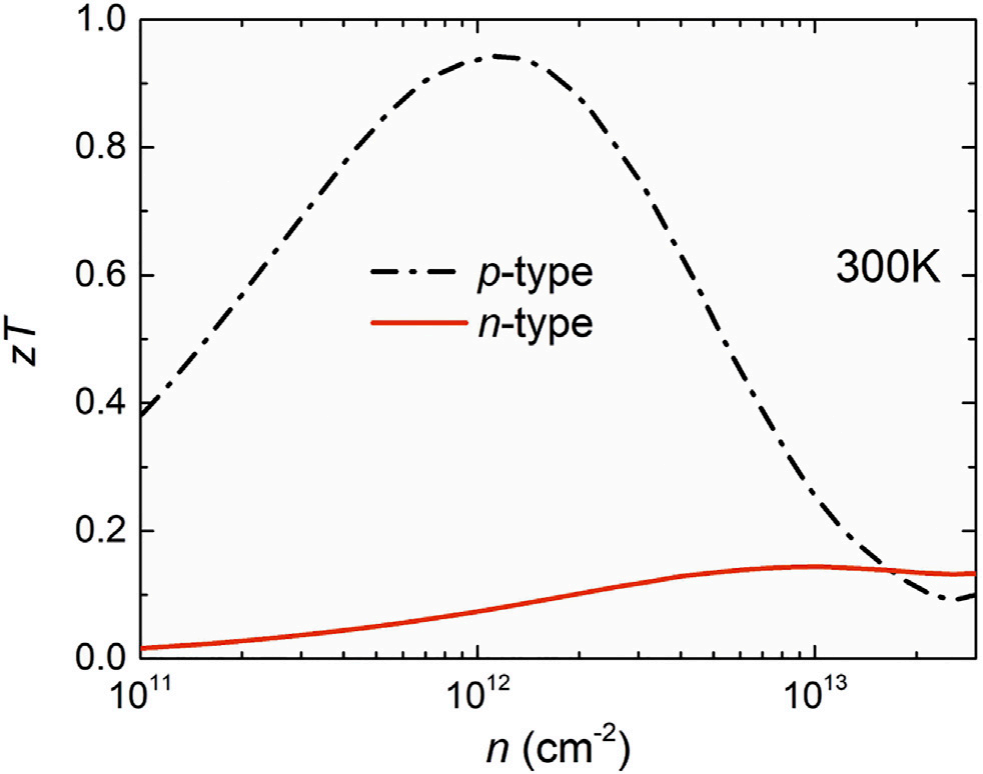
Calculated thermoelectric figure of merit at 300 K.

Combining the electronic and phonon transport properties, we evaluated the thermoelectric performance of the $\beta_{1}^{s}$ boron monolayer. Figure 5 shows the figure of merit *ZT* values for both the *n-* and *p*-doped boron monolayer as a function of the carrier concentration at room temperature. Obviously, the *p*-type thermoelectric performance is superior to *n*-type due to the excellent *p*-type power factor. Combined with the relatively low lattice thermal conductivity, the optimal *p*-type *ZT* value of the boron monolayer reaches 0.96 at an optimal carrier concentration of about 1 × 10^12^ cm^−2^, which is a recorded value among single-layer materials consisting of light elements. Our results indicate that the semiconducting $\beta_{1}^{s}$ boron monolayer has a potential application in thermoelectric devices.

## Conclusion

We have investigated the thermoelectric performance of a semiconducting $\beta_{1}^{s}$ boron monolayer using first-principles combined with Boltzmann transport equations. We have shown that the high *n*-type Seebeck coefficient arises from the camelback shape of the lowest conduction band, while the highly degenerate valence band with small effective mass leads to the high hole mobility and long relaxation time, which contributes to the superior hole transport performance. Importantly, we found relatively low lattice thermal conductivity in the boron monolayer, ∼20 W/mK at 300 K, as compared with graphene or phosphorene also consisting of a light element. This is primarily ascribed to the complex unit cell with the hexagonal vacancy. Finally, we obtained an optimal *p*-type *ZT* of about 0.96 at 300 K in this boron monolayer, indicating its potential as *p*-type thermoelectric materials.

## Data Availability

The original contributions presented in the study are included in the article/supplementary material; further inquiries can be directed to the corresponding author.
